# Presynaptic SNAP25 supports maturation of hippocampal mossy fiber-CA3 synapses

**DOI:** 10.1016/j.isci.2026.116503

**Published:** 2026-06-20

**Authors:** Shuichi Hayashi, Nobuhiko Ohno, Zoltán Molnár, Kazunori Toida

**Affiliations:** 1 Department of Anatomy, Kawasaki Medical School, 577 Matsushima, Kurashiki, Okayama 701-0192, Japan; 2 Department of Anatomy, Division of Histology and Cell Biology, School of Medicine, Jichi Medical University, 3311-1 Yakushiji, Shimotsuke, Tochigi 329-0498, Japan; 3 Division of Ultrastructural Research, National Institute for Physiological Sciences, 5-1 Higashiyama Myodaiji, Okazaki, Aichi 444-8787, Japan; 4 Department of Physiology, Anatomy and Genetics, Sherrington Building, University of Oxford, Parks Road, Oxford OX1 3PT, UK

**Keywords:** molecular neuroscience, cellular neuroscience

## Abstract

Mossy fiber (MF)-CA3 synapses in the hippocampus play vital roles in learning and memory. MFs have characteristic giant boutons with thorny excrescences on the dendrites of CA3 pyramidal neurons. The mechanisms underlying the development of this complex synaptic specialization remain unclear. In the present study, the loss of synaptosomal-associated protein 25 (SNAP25)—a protein essential for regulated synaptic vesicular release—increased the density but decreased the size of MF boutons and altered the postsynaptic distribution of homer scaffolding protein 1. Three-dimensional correlative light and electron microscopy revealed that although axon targeting and synapse formation were unaffected, excrescences failed to develop in MF boutons, resulting in a smaller contact area between MF boutons and CA3 dendrites. Moreover, SNAP25-deficient boutons displayed abnormal intracellular profiles, such as the accumulation of large synaptic vesicles. These findings indicate that presynaptic SNAP25 is essential for the maturation and maintenance of specialized hippocampal giant boutons.

## Highlights

• SNAP25 is dispensable for mossy fiber targeting in the hippocampus
• SNAP25-deficient mossy fiber boutons fail to mature in postnatal development
• Presynaptic SNAP25 loss impairs postsynaptic thorny excrescence formation
• Loss of SNAP25 leads to abnormal synaptic vesicle accumulation in adults

## Graphical abstract

**Figure:**
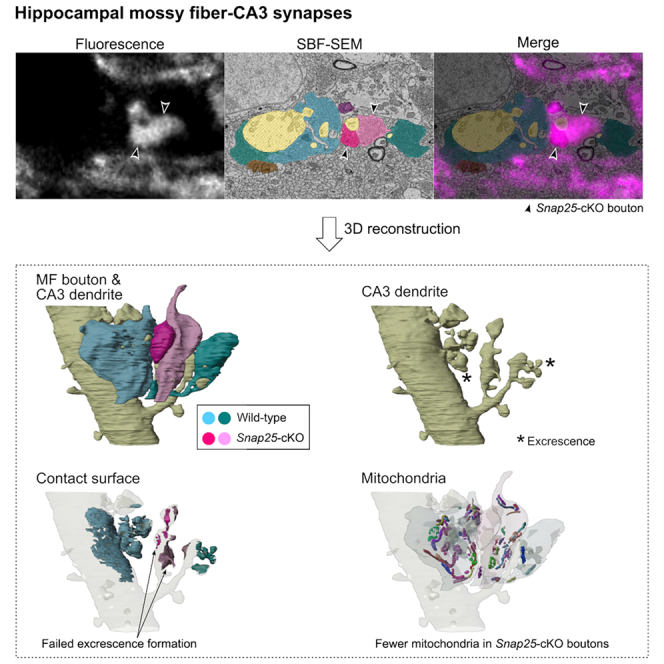

## Introduction

The hippocampus plays a central role in higher cognitive functions of the brain, including memory, learning, and spatial navigation.^1^^,^^2^ Synaptic morphogenesis is among the most important processes underlying these functions and is controlled by various factors, including cell surface proteins, signaling molecules, and neuronal activity.^3^^,^^4^^,^^5^ However, the mechanisms underlying the development and maintenance of hippocampal synapses remain unclear.

Mossy fibers (MFs) originate from granule cells in the dentate gyrus and constitute strong feedforward connections within the hippocampal “canonical trisynaptic loop.” MFs form characteristic giant boutons that connect with the proximal part of CA3 pyramidal neuron apical dendrites in the stratum lucidum.^6^ Each MF bouton envelops multiple spiny structures, known as “thorny excrescences,” that emerge from CA3 dendrites, on which multiple synapses are formed.^7^ Unlike the single-contact synapses that are widely observed in the brain, such as those in the cerebral cortex and hippocampal CA1, complex synaptic connections with excrescences are observed in relatively limited areas, typically in the thalamus; specifically, in the dorsolateral geniculate nucleus and posterior nucleus (Po), which receive retinal (retinogeniculate) inputs and corticothalamic inputs from layer 5b, respectively.^8^ The complex MF-CA3 synaptic connections are established by sequential morphological changes in both presynaptic boutons and postsynaptic dendrites during the first few postnatal weeks^9^^,^^10^^,^^11^; this resembles the development of retinogeniculate^12^ and cortical layer 5b-Po synapses.^13^ First, each MF bouton forms a single synapse with CA3 dendrites by the end of the first postnatal week. Both MF bouton size and complexity markedly increase between postnatal day (P)7 and P14 in mice and rats, and thorny excrescences protrude from the dendrites into the MF bouton to form multiple synapses. Further increases in MF bouton size occur, and the refinement of excrescences takes place after P21, reaching a specialization similar to that of adults at P28. Moreover, the subpopulation of MF boutons continues to grow throughout life.^14^ MFs provide powerful inputs to CA3 neurons through giant boutons, and MF-CA3 synapses exhibit various forms of short- and long-term plasticity.^15^^,^^16^^,^^17^ Those unique transmission and plasticity properties of MF-CA3 synapses are based on their complex structures and biophysical and molecular properties.^18^ Deciphering the regulatory mechanisms underlying synaptic morphogenesis will, therefore, be the key to understanding how functional hippocampal networks are established during development.

Synaptosomal-associated protein 25 (SNAP25) is an SNARE (soluble N-ethylmaleimide-sensitive factor attachment protein receptor) complex protein that is required for the fusion of synaptic vesicles to release neurotransmitters at the presynapse.^19^^,^^20^^,^^21^ SNAP25 has been associated with various psychiatric and neurological disorders, including schizophrenia^22^^,^^23^ and attention-deficit/hyperactivity disorder.^24^^,^^25^^,^^26^ To understand the mechanisms underlying human disorders associated with SNAP25 dysfunction, a functional analysis of SNAP25 is required. *Snap25*-null mice exhibit lethality at birth,^19^ which hinders our understanding of the effects of *Snap25* removal on postnatal brain development. However, our previous studies using the conditional knockout of *Snap25* with cortical layer-specific Cre drivers have revealed that removing *Snap25* in layer 5, layer 6a, or layer 6b neurons of the cerebral cortex does not affect the initial targeting, synapse formation, and myelination of axonal projections.^27^^,^^28^ In addition, the cortical layer 5-specific knockout of *Snap25* does not affect the initial synaptic assembly of corticothalamic projections in the Po. However, *Snap25*-deleted layer 5 corticothalamic projections fail to develop mature complex synaptic structures; they have smaller boutons and no excrescence from Po dendrites forming into the boutons.^13^ These mice exhibit increased wakefulness,^29^ suggesting the importance of the cerebral cortex in sleep-wake regulation. Moreover, in the absence of *Snap25*, axons from cortical layer 5 begin to degenerate after P21; this is evident in tracts including the corticospinal, corticothalamic, and callosal projections.^27^

Given that MF-CA3 synapses have a complex synaptic specialization that is similar to that of cortical layer 5-Po connections, SNAP25 may regulate the development and maintenance of MF-CA3 synapses in the hippocampus. Despite this hypothesis, previous studies have indicated that *Snap25*-deleted MFs do not exhibit any significant morphological abnormalities or degenerative characteristics at the light microscopic level.^27^^,^^30^ Moreover, the elimination of glutamatergic synaptic transmission does not affect synaptic development in the hippocampus, including that of MF-CA3.^31^^,^^32^^,^^33^ Does the developmental mechanism for MF boutons differ from that for boutons of layer 5 corticothalamic projections? To address this question, the present study examined the effects of *Snap25* knockout on MF-CA3 synapses. To ablate SNAP25 from a selected population of neurons in the dentate gyrus, we used *Rbp4-Cre* mice, in which a subset of neurons in the dentate gyrus express Cre from around embryonic day (E) 16.5 onward,^27^^,^^34^^,^^35^ and crossed them with *Snap25*-floxed mice. We then used three-dimensional (3D) correlative light and electron microscopy (CLEM), which involves correlating confocal microscopy images of MF boutons with images obtained using volume electron microscopy (EM) without immunostaining. Our 3D ultrastructural analyses demonstrated that presynaptic SNAP25 is essential for both presynaptic and postsynaptic morphogenesis in MF-CA3 synapses, particularly in the formation of thorny excrescences. Moreover, in mice with conditional SNAP25 ablation, SNAP25-ablated boutons exhibited dark cytoplasm and the accumulation of large synaptic vesicles, thus indicating disrupted presynaptic vesicular homeostasis and possible synaptic degeneration. Together, these results suggest that synaptic development and the maintenance of giant boutons in MF-CA3 and cortical layer 5-Po connections share a common SNAP25-dependent mechanism.

## Results

### Excrescence formation is impaired in SNAP25-deficient boutons

**Figure 1:**
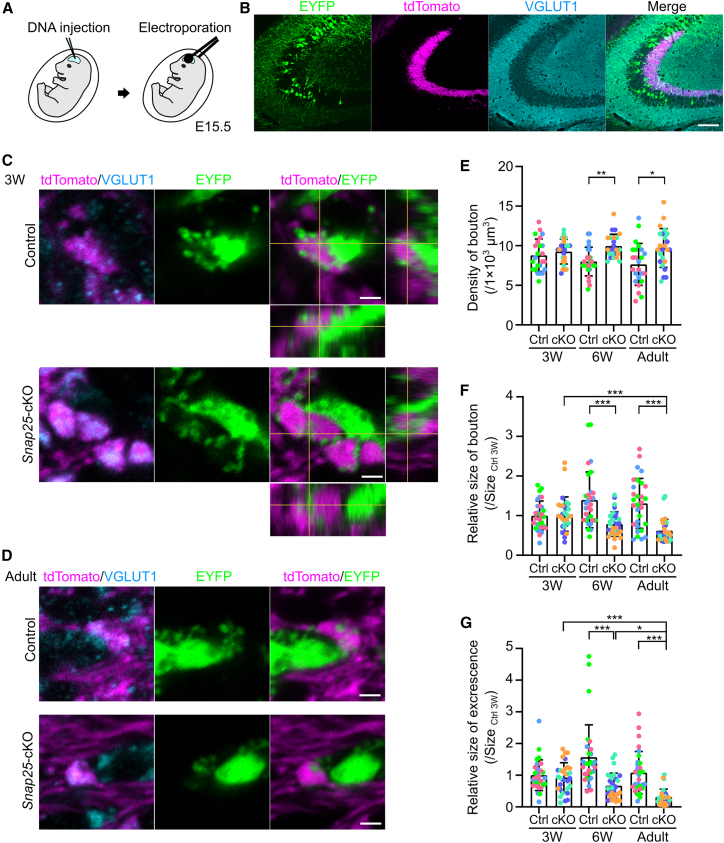
Impaired formation of thorny excrescences into SNAP25-deficient boutons (A) Schematic of *in utero* electroporation (IUE) for labeling CA3 dendrites. DNA solution was injected into the left lateral ventricle of embryos at E15.5, and electric pulses were applied to introduce plasmids into the hippocampal formation. (B) Typical example of the CA3 region in the hippocampus, where pyramidal neurons express electroporated EYFP, at P21. MFs were positive for VGLUT1, and some were labeled with *Rbp4-Cre*-driven tdTom fluorescence. (C and D) Examples of MF boutons that contacted CA3 dendrites labeled with EYFP at P21 (C) and in adulthood (D). Thorny excrescences from the connecting CA3 dendrite protruded into the control MF bouton (top). Dendritic protrusions only contacted the outside of the connecting *Snap25*-cKO boutons (bottom). (E–G) The density (E) and size (F) of tdTom^+^ boutons and the size of their contacting excrescences labeled with EYFP (G) were compared between the control and *Snap25*-cKO mice at the age of 3 weeks (w), 6 w, and in adulthood. The size of boutons at each stage of control and *Snap25*-cKO brains relative to the mean size of boutons in control brains at 3 w of age (/size _Ctrl 3w_) is shown in (F). The size of excrescences at each stage of control and *Snap25*-cKO brains relative to the mean size of excrescences in control brains at 3 w of age (/size _Ctrl 3w_) is shown in (G). The data in the bar graphs are represented as mean ± SD with all data points. Data from different animals are shown in different colors. There were significant differences between the control and *Snap25*-cKO groups at 6 w of age and in adulthood for all parameters. The size of boutons and excrescences in *Snap25*-cKO mice in adulthood was significantly smaller than that at 3 w of age. Kruskal-Wallis test followed by post hoc Dunn’s multiple-comparison test; ∗*p* < 0.05, ∗∗*p* < 0.01, ∗∗∗*p* < 0.001. Scale bars: 10 μm (B) and 2.5 μm (C and D). See also Figure S1.

To understand the involvement of SNAP25 in the formation of MF-CA3 synapses during postnatal development, we labeled MFs with tdTomato (tdTom) fluorescence by crossing Ai14 reporter mice with *Rbp4-Cre* mice, which express Cre in a subset (approximately 40%) of granule cells of the dentate gyrus (Figures S1A and S1B).^29^^,^^36^ Because no specific antibody was available to label CA3 pyramidal neurons—the recipient cells of MF input—we used *in utero* electroporation to express enhanced yellow fluorescent protein (EYFP) in CA3 pyramidal neurons. A plasmid vector encoding *Eyfp* was injected into one side of the lateral ventricle of embryos at E15.5, followed by electric pulses to introduce the plasmids into the hippocampal formation (Figures 1A, S1C, and S1D). The electroporated vector was mainly expressed by CA1–CA3 neurons in the hippocampus (Figure 1B). *Rbp4-Cre*-driven tdTom fluorescence labeled MFs, and their boutons were positive for the presynaptic marker vesicular glutamate transporter 1 (VGLUT1) (Figure 1B). To understand the role of SNAP25 in the postnatal development of MF-CA3 synapses, we crossed *Snap25*-floxed mice with *Rbp4-Cre;Ai14* mice and examined the development of MF-CA3 synapses. We confirmed the loss of SNAP25 staining on MFs at P21 in our previous study^27^ and also at 6 weeks of age in the present study (Figure S1E). Because *Rbp4-Cre* expression in the granule cells of the dentate gyrus was barely detectable at P2 but detectable by P8,^13^^,^^27^^,^^36^ *Snap25* was assumed to be conditionally knocked out (*Snap25*-cKO) by the end of the first postnatal week. Given that the major morphological changes of MF-CA3 synapses occur between P7 and P14, and their growth and refinement continue after P21,^9^^,^^10^ we examined the development of MF-CA3 synapses at P21, P42 (i.e., 6 weeks of age), and 3–4 months of age (considered an adult time point). Electroporated EYFP in CA3 pyramidal neurons labeled their apical dendrites and dendritic excrescences extending into MF boutons (Figure 1C, EYFP). When we compared tdTom-positive (tdTom^+^) MF boutons and their contacting CA3 dendrites at 3 weeks of age, the excrescences overlapped with tdTom^+^ boutons in *Rbp4-Cre*;*Snap25*(*+/f*) brains (control) (Figure 1C, top), whereas dendritic excrescences mainly contacted the outside of tdTom^+^ boutons in *Rbp4-Cre*;*Snap25*(*f/f*) brains (i.e., those of *Snap25*-cKO mice) (Figure 1C, bottom). In 6-week-old (Figure S1F) and adult (3–4 months, Figure 1D) brains, fewer or no dendritic excrescences were observed to contact the tdTom^+^ boutons. The density of tdTom^+^ boutons in the CA3 (per 1 × 10^3^ μm^3^) was 8.8 ± 2.0 in control mice and 9.3 ± 1.6 in *Snap25*-cKO mice at 3 weeks of age, 8.0 ± 1.8 in control mice and 10.0 ± 1.5 in *Snap25*-cKO mice at 6 weeks of age, and 7.6 ± 2.7 in control mice and 9.7 ± 2.5 in *Snap25*-cKO mice in the adults (mean ± SD, *n* = 24 areas from three brains for each genotype at different ages; Figure 1E). There was no significant difference between the control and *Snap25*-cKO brains at 3 weeks of age (*p* > 0.99). By contrast, the density of tdTom^+^ boutons was significantly higher in *Snap25*-cKO mice than in control mice at 6 weeks of age (*p* < 0.01) and in adulthood (*p* < 0.05). We also compared the size of boutons and excrescences that contacted tdTom^+^ boutons; this value was normalized to the value in control brains at 3 weeks of age. This relative size of MF boutons in *Snap25*-cKO mice at 3 weeks of age was 1.0 ± 0.4, which was not significantly different from that of the control (1.0 ± 0.4) (mean ± SD, *n* = 30 from three brains for each genotype at different ages, *p* > 0.99; Figure 1F). By contrast, the relative size of MF boutons was significantly smaller in *Snap25*-cKO mice than in control mice at 6 weeks of age (control: 1.4 ± 0.7, *Snap25*-cKO: 0.8 ± 0.3, *p* < 0.001) and at the adult time point (control: 1.3 ± 0.6, *Snap25*-cKO: 0.6 ± 0.3, *p* < 0.001). Moreover, in *Snap25*-cKO mice, the relative size of MF boutons was significantly smaller in adult mice than in mice at 3 weeks of age (*p* < 0.001), indicating that the phenotype becomes more severe from late postnatal development to young adulthood. Similar differences were observed in the relative size of excrescences at 3 weeks (control: 1.0 ± 0.5, *Snap25*-cKO: 0.9 ± 0.5, *p* > 0.99) and 6 weeks (control: 1.5 ± 1.0, *Snap25*-cKO: 0.7 ± 0.4, *p* < 0.001) of age, and in adulthood (control: 1.1 ± 0.7, *Snap25*-cKO: 0.3 ± 0.2, *p* < 0.001) (*n* = 30 from three brains for each genotype at different ages; Figure 1G). Together, these results indicate that presynaptic SNAP25 is required for the development of MF boutons and CA3 excrescences, as well as for pruning in the hippocampus.

### Altered postsynaptic protein assembly in the absence of presynaptic SNAP25

**Figure 2:**
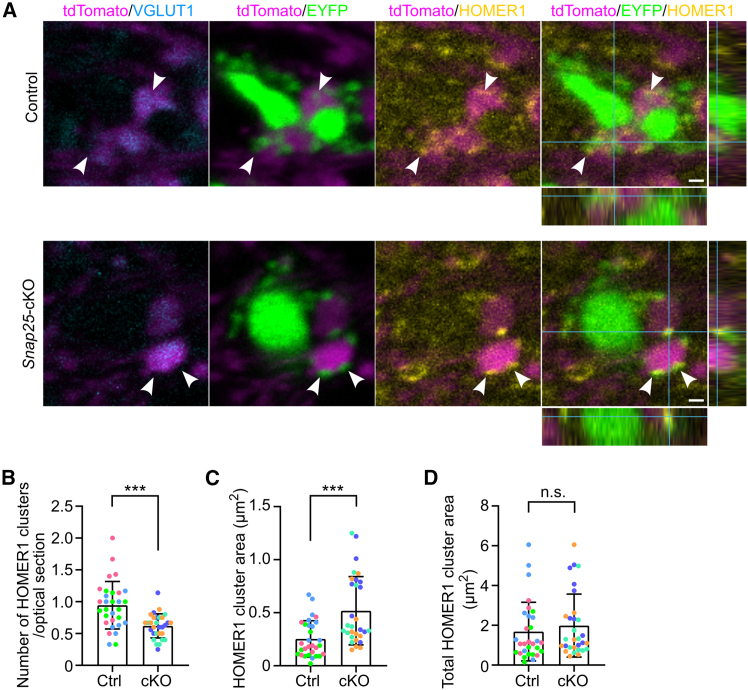
Recruitment of HOMER1 to the contact sites between MF boutons and CA3 dendrites is independent of SNAP25 (A) HOMER1 was localized at the excrescences contacting MF boutons in both control and *Snap25*-cKO mice at six weeks of age; however, its distribution in *Snap25*-cKO mice was more restricted than that in control mice. Arrows in orthogonal views indicate examples of HOMER1 localization. Scale bars: 1 μm. (B–D) Compared with the findings in control mice, the number of HOMER1 clusters (B) was significantly smaller, but the area of the clusters (C) was significantly greater in *Snap25*-cKO mice. No significant difference was observed in total HOMER1 cluster area between the two groups (D). The data in the bar graphs are represented as mean ± SD with all data points. Data from different animals are shown in different colors. Mann-Whitney *U* test; ∗∗∗*p* < 0.001. See also Figure S2.

To determine whether synapses were formed in *Snap25*-cKO boutons, we examined the distribution of the presynaptic marker Bassoon and the postsynaptic marker homer scaffolding protein 1 (HOMER1) at 6 weeks of age. Bassoon and HOMER1 localized at the contact sites between tdTom^+^ boutons and EYFP-labelled dendrites both in control and *Snap25*-cKO brains (Figure S2A). These results suggest that the assembly of synaptic proteins itself is not affected by *Snap25*-cKO. However, the HOMER1 clusters in *Snap25-*cKO brains were more confined than those in control brains (Figure 2A). We, therefore, quantified the area of HOMER1 clusters at the contact sites between tdTom^+^ boutons and EYFP-labelled dendrites (Figures S2B and S2C). The mean number of clusters on bouton per optical section was 0.94 ± 0.37 in control boutons and 0.61 ± 0.18 in *Snap25*-cKO boutons (mean ± SD, *n* = 30 boutons from three brains), and the former was significantly larger than the latter (*p* < 0.001, Figure 2B). By contrast, the size of the clusters was 0.25 ± 0.17 μm^2^ in control boutons and 0.52 ± 0.32 μm^2^ in *Snap25*-cKO boutons, and the former was significantly smaller than the latter (*p* < 0.001, Figure 2C). Despite the reduced number of clusters in *Snap25-*cKO boutons, their larger cluster area per bouton resulted in no significant difference in the total HOMER1 cluster area per bouton between the control (1.7 ± 1.5 μm^2^) and *Snap25*-cKO (2.0 ± 1.6 μm^2^) groups (*p* = 0.36; Figure 2D). These findings indicate that, although synaptic assembly itself does not depend on SNAP25, the failure of excrescence formation alters the contact area between MF boutons and CA3 dendrites, thereby affecting synaptic protein distribution in dendrites.

### MF bouton assembly on CA3 dendrites is not altered in the absence of SNAP25

**Figure 3:**
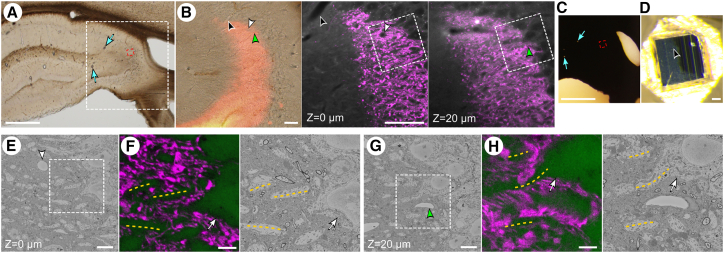
CLEM workflow to identify tdTom^+^ MF boutons in electron micrographs (A) Bright-field image of a *Snap25*-cKO hippocampal slice. The white dotted box indicates the region shown in (C). Red boxes in (A) and (C) indicate the regions that were focused on using confocal microscopy and SBF-SEM. The light blue arrows in (A) and (C) indicate the blood vessels that were used to correlate the bright-field image with the image after embedding in resin (C). (B) Overlay of bright-field and fluorescence images of the CA3 region (left) and confocal images of the slice surface (middle) and a region 20 μm deeper (right). In the confocal images, background signal was used to visualize the positions of blood vessels and cell bodies (gray), and the image was overlaid with tdTom fluorescence (magenta). (C) Part of the slice embedded in resin, corresponding to the white dotted box in (A). Light blue arrows indicate the blood vessels that were used for correlation. (D) The embedded slice was placed on a rivet and trimmed further. The blood vessel corresponding to that shown in (B) is indicated by a black arrow. (E–H) SBF-SEM images of the surface area of the slice (E and F) and a region 20 μm deeper (G and H). Parts of the fluorescence images (left images in F and H) and SBF-SEM (right images in F and H) were used to correlate the stack of fluorescence images with that of SBF-SEM. The fluorescence signals of the images capturing the background were inverted and pseudo-colored green to identify the neuronal cell bodies, dendrites, and blood vessels. Scale bars: 0.5 mm (A and C), 100 μm (D), 50 μm (B), 10 μm (E and G), and 5 μm (F and H).

**Figure 4:**
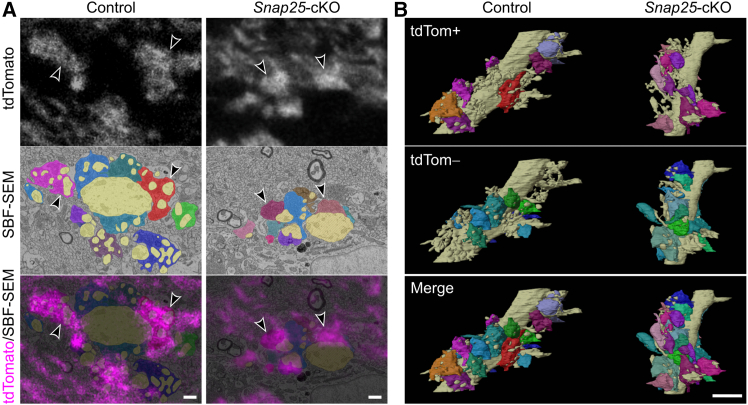
Distribution of MF boutons on CA3 dendrites (A) CLEM of fluorescence and SBF-SEM images of the CA3 region in the hippocampus. Individual MF boutons on the same dendrite are indicated by different colors in the SBF-SEM images, and representative tdTom^+^ boutons are indicated by black arrowheads. (B) 3D reconstruction of boutons on single dendrites in control (left) and *Snap25*-cKO (right) brains. The distributions of tdTom^+^ (top), tdTom^−^ (middle), and both (bottom) boutons are shown. Scale bars: 1 μm (A) and 5 μm (B).

To gain a deeper understanding of the structural defects induced by *Snap25*-cKO in MF-CA3 synapses, we examined their ultrastructure. Because *Rbp4-Cre* is not expressed by all granule cells in the dentate gyrus, we used CLEM to identify Cre-driven tdTom^+^ boutons in the CA3 region.^37^^,^^38^^,^^39^ Using this method, Cre-driven tdTom fluorescence was observed without immunostaining to achieve the high-quality ultrastructural preservation of tissue. Throughout sample preparation and microscopy observation, the correlation of images obtained before and after embedding was crucial for identifying tdTom^+^ boutons in electron micrographs. First, bright-field and fluorescence images of hippocampal slices were captured. Natural landmarks on the slices, such as blood vessels (Figures 3A and 3B, arrows and arrowheads), were used to correlate these images with those obtained post-embedding (Figures 3C and 3D) and those acquired using serial block-face scanning EM (SBF-SEM; Figures 3E–3H). Blood vessels that extended from the surface to the deeper CA3 region in the slice were particularly useful for correlation at the beginning of SBF-SEM imaging (Figures 3B [middle], 3E, and 3F). Once the region of interest was set, SBF-SEM images were acquired at a higher resolution. During and after SBF-SEM imaging, we verified the slice planes by matching the obtained image stacks with the confocal images taken before embedding and by aligning them with the corresponding SBF-SEM image planes (Figures 3F and 3H). Further correlation was conducted using higher-magnification confocal images to identify tdTom^+^ boutons on SBF-SEM images (Figure 4A). This correlative method allowed us to simultaneously identify tdTom^+^ (*Snap25*-heterozygous in control brains and *Snap25*-homozygous in *Snap25*-cKO brains) and tdTom-negative (tdTom^−^; *Snap2*5-wild-type in both control and *Snap2*5-cKO brains) boutons. To determine whether the distribution of *Snap25*-cKO boutons was altered, we focused on MF boutons that were distributed on single dendrites in the control and *Snap25*-cKO groups. The 3D reconstruction of segmented MF-boutons and their connecting dendrites demonstrated that tdTom^+^ and tdTom^−^ boutons intermingled along the dendrites in both control and *Snap25*-cKO mice (Figure 4B). These results indicated that the targeting of MFs to the proximal dendrites of CA3 neurons is not affected by the loss of SNAP25.

### Excrescence formation depends on presynaptic SNAP25

**Figure 5:**
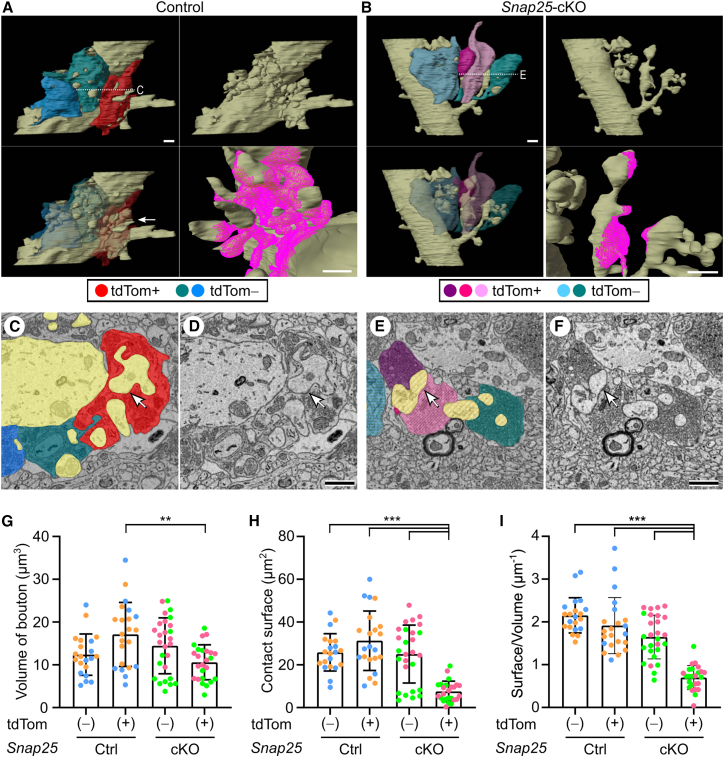
Decreased volume and contact surface area of *Snap25*-cKO boutons (A and B) 3D reconstruction model of single dendrites with (top left and bottom left) and without (top right and bottom right) connecting MF boutons. These are parts of the models in Figure 4B. Some dendritic branches and excrescences, which did not contact the MF boutons shown in (A) and (B), are omitted. Boutons with increased transparency are shown in the lower left. The control (*Snap25*-heterozygous) bouton indicated by red contacts with excrescences (mesh in the lower right of A), whereas the SNAP25-deficient bouton (*Snap25*-homozygous) indicated by light pink contacts only with the shaft of the dendritic branch (mesh in the lower right of B). The bouton indicated by purple is not visible from the front view in (B) (see the section in E). The dotted lines with the letter C in (A) and letter E in (B) indicate the planes of images shown in Figures 5C and 5E, respectively. (C–F) Typical example of single sections in control (C and D) and *Snap25*-cKO (E and F) brains. The plane is shown by dotted lines in (A) and (B). Arrows indicate synapses. (G–I) Volume of MF boutons (G), their contact surface area with the connecting excrescences or dendritic shafts (H), and the ratio of the contact surface area to the bouton volume (I). The data in the bar graphs are represented as mean ± SD with all data points. Data from different animals are shown in different colors. The volume of tdTom^+^ boutons (*Snap25*-homozygous) in *Snap25*-cKO brains was significantly smaller than that of tdTom^+^ boutons (*Snap25*-heterozygous) in control brains but was not significantly different from that of tdTom^−^ boutons (*Snap25*-wild-type) in control or *Snap25*-cKO brains. The contact surface area and its ratio to the bouton volume of tdTom^+^ boutons with their contacting excrescences in *Snap25*-cKO brains were significantly smaller than those of other boutons. Kruskal-Wallis test followed by post hoc Dunn’s multiple-comparison test; ∗∗*p* < 0.01, ∗∗∗*p* < 0.001. Scale bars: 1 μm.

The 3D reconstruction of segmented boutons and their connecting dendrites revealed that each bouton in the control group enveloped one to three excrescences stemming from the contacting dendrites (Figure 5A). By contrast, SNAP25-deficient boutons only contacted dendritic shafts or branches of CA3 pyramidal neurons (Figure 5B), consistent with the confocal microscopy results (Figures 1C and 1D, bottom). Synaptic vesicles accumulated at the contact sites between each MF bouton and at the connecting excrescences in the control group (Figures 5C and 5D, arrows), and even SNAP25-deficient boutons appeared to form synapses with dendritic shafts (Figures 5E and 5F, arrows). This observation is consistent with the observation of HOMER1 assembly at the contact sites between SNAP25-deficient boutons and dendrites (Figure 2), indicating that the initial synaptic assembly is not dependent on SNAP25. We then examined the bouton volume and contact surface between MFs and connecting dendrites, using 3D reconstruction models in control and *Snap25*-cKO brains. In control brains, the volumes of tdTom^−^ (*Snap25*-wild-type) and tdTom^+^ (*Snap25*-heterozygous) boutons were 12.4 ± 4.8 and 17.1 ± 7.5 μm^3^, respectively, whereas in *Snap25*-cKO brains, the volumes of tdTom^−^ (*Snap25*-wild-type) and tdTom^+^ (*Snap25*-homozygous) boutons were 14.5 ± 6.5 and 10.6 ± 4.1 μm^3^, respectively (mean ± SD, *n* = 21, 22, 27, and 25 boutons from two brains for the tdTom^−^ and tdTom^+^ populations in each of control and *Snap25*-cKO brains, respectively; Figure 5G). The tdTom^+^ boutons in *Snap25*-cKO (*Snap25*-homozygous) mice were significantly smaller than those in control (*Snap25*-heterozygous) mice (*p* < 0.01) but were not significantly different from the tdTom^−^ boutons (*Snap25*-wild-type) in the control and *Snap25*-cKO brains (*p* > 0.99 and *p* = 0.18, respectively). The contact surface areas between MF boutons and CA3 dendritic branches or excrescences of tdTom^−^ and tdTom^+^ boutons in control brains were 25.9 ± 8.7 and 31.3 ± 13.9 μm^2^, respectively, whereas in *Snap25*-cKO brains, the contact surface areas of tdTom^−^ and tdTom^+^ boutons were 25.1 ± 13.5 and 7.6 ± 4.8 μm^2^, respectively (mean ± SD, *n* = 21, 22, 27, and 25 boutons from two brains for the tdTom^−^ and tdTom^+^ populations in each of control and *Snap25*-cKO brains, respectively; Figure 5H). The contact surface area of tdTom^+^ boutons in *Snap25*-cKO mice was significantly smaller than those of tdTom^−^ and tdTom^+^ boutons in the other groups (*p* < 0.001). Because the volume of tdTom^+^ boutons was also decreased in *Snap25*-cKO mice, we compared the ratio of the contact surface area to the bouton volume; this ratio was significantly smaller in tdTom^+^ boutons in *Snap25*-cKO mice than in tdTom^−^ and tdTom^+^ boutons in the other groups (*p* < 0.001, Figure 5I). These results indicated that the reduced contact surface area of SNAP-deficient boutons was mainly caused by an absence of excrescences, rather than a decreased bouton volume. Importantly, even when excrescences failed to form in *Snap25*-deficient boutons, they formed normally in adjacent *Snap25*-wild-type boutons that interacted with the same dendrites (Figures 4B, 5B–5E, and 5F). Because *Snap25* was deleted in presynaptic MFs but not in postsynaptic CA3 dendrites, these results indicate that postsynaptic dendritic morphogenesis is dependent on presynaptic inputs via SNAP25.

### Alteration of cytoplasm and synaptic vesicles in SNAP25-deficient boutons

**Figure 6:**
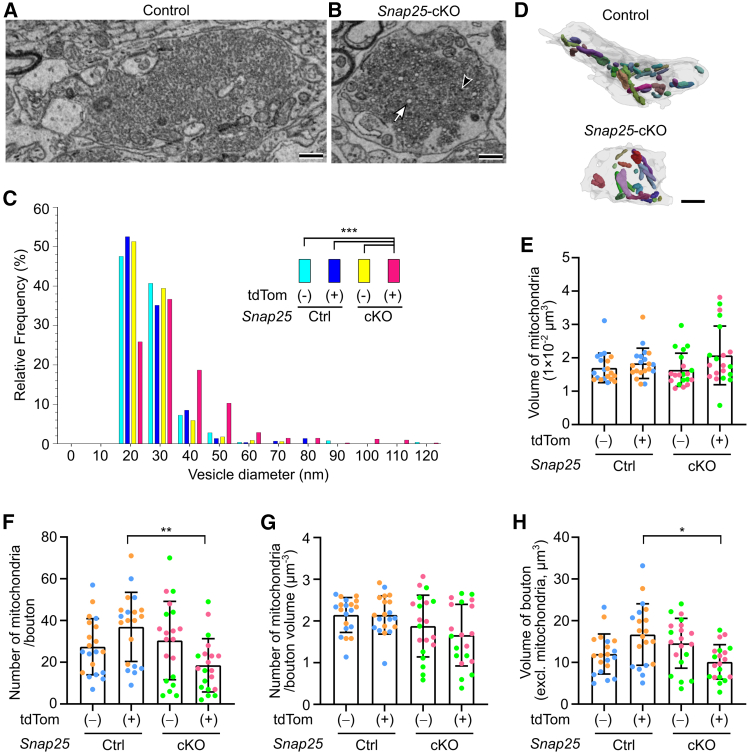
*Snap25*-cKO boutons have dark cytoplasm and decreased mitochondria (A and B) SBF-SEM images of control (A) and *Snap25*-cKO (B) boutons. White arrows and black arrowheads indicate large vesicles and dark spots in the cytoplasm, respectively. (C) Relative frequency of vesicle diameters in MF boutons of control and *Snap25*-cKO brains. The labels on the *x* axis indicate the center of each bin. The vesicle diameter was significantly larger in tdTom^+^ boutons (*Snap25*-homozygous) of *Snap25*-cKO brains than in tdTom^+^ and tdTom^−^ boutons of the other groups. (D) Typical 3D models of mitochondria in tdTom^+^ boutons in control (upper) and *Snap25*-cKO (lower) brains. (E–H) Volume of mitochondria (E), number of mitochondria per bouton (F), number of mitochondria per bouton volume (G), and volume of boutons excluding mitochondrial volume (H). The data in the bar graphs are represented as mean ± SD with all data points. Data from different animals are shown in different colors. No significant difference was observed in the volume of mitochondria in each bouton (E), whereas the number of mitochondria was significantly lower in tdTom^+^ boutons in *Snap25*-cKO brains than in tdTom^+^ boutons in control brains (F). No significant differences were observed in the number of mitochondria per bouton volume among the genotypes (G). The volume, excluding that of mitochondria, of tdTom^+^ boutons in *Snap25*-cKO brains was significantly smaller than that of control brains (H). Kruskal-Wallis test followed by post hoc Dunn’s multiple-comparison test; ∗*p* < 0.05, ∗∗*p* < 0.01, ∗∗∗*p* < 0.001. Scale bars: 500 nm (A and B) and 1 μm (D).

Fluorescence microscopy observations of MFs did not show any detectable signs of axonal degeneration, such as fragmentation, which has been observed in corticothalamic projections from cortical layer 5 into the Po and corticospinal tracts.^27^ However, on SBF-SEM images of hippocampal CA3, we observed that *Snap25*-cKO boutons often contained dark spots and vesicles that were larger than those in control boutons (Figures 6A and 6B, arrowhead and arrow). These characteristics were sufficiently distinct to allow distinguishing SNAP25-deficient boutons from intact boutons during SBF-SEM imaging, even before analyzing their correlation with confocal images. The mean diameters of synaptic vesicles in tdTom^−^ and tdTom^+^ boutons in control brains were 27.8 ± 10.5 nm (*n* = 248 vesicles) and 27.4 ± 9.4 nm (*n* = 293 vesicles), respectively, and those in tdTom^−^ and tdTom^+^ boutons in *Snap25*-cKO were 26.3 ± 7.5 nm (*n* = 337 vesicles) and 35.3 ± 16.3 nm (*n* = 417 vesicles), respectively (mean ± SD from two brains for each genotype; Figure 6C). The size of vesicles in tdTom+ boutons in *Snap25*-cKO brains (SNAP25-deficient boutons) was significantly larger than that in tdTom^−^ and tdTom^+^ boutons in the other groups (*p* < 0.001). These results suggest that despite the absence of clear degenerative features at the fluorescence microscopic level, intracellular alterations occur with the loss of SNAP25 in MF boutons.

We also examined mitochondria in MF boutons (Figure 6D) because their profiles change in neurodegenerative diseases such as Alzheimer disease.^40^^,^^41^^,^^42^ The mean mitochondrial volume per bouton (1 × 10^−2^ μm^3^) was not significantly different between tdTom^+^ and tdTom^−^ boutons in control brains (tdTom^−^: 1.7 ± 0.4, tdTom^+^: 1.8 ± 0.5) and those in *Snap25*-cKO brains (tdTom^−^: 1.6 ± 0.5, tdTom^+^: 2.0 ± 0.9) (*n* = 20 from two brains for each genotype, *p* = 0.10; Figure 6E). In control brains, the numbers of mitochondria in tdTom^−^ and tdTom^+^ boutons were 27.5 ± 13.5 and 37.0 ± 16.6, respectively, whereas in *Snap25*-cKO brains, the numbers of mitochondria in tdTom^−^ and tdTom^+^ boutons were 30.4 ± 18.8 and 18.6 ± 12.8, respectively (mean ± SD, *n* = 20 boutons from two brains for each genotype). There were significantly fewer mitochondria per bouton in tdTom^+^ boutons of *Snap25*-cKO brains than in tdTom^+^ boutons of control brains (*p* < 0.01, Figure 6F). Considering the reduced volume of tdTom^+^ boutons in *Snap25*-cKO mice, we compared the numbers of mitochondria relative to bouton volume (μm^−3^) and identified no significant difference among the genotypes (tdTom^−^: 2.1 ± 0.4 and tdTom^+^: 2.1 ± 0.5 in control brains; tdTom^−^: 1.8 ± 0.7 and tdTom^+^: 1.7 ± 0.7 in *Snap25*-cKO brains, *p* = 0.13; Figure 6G). These results indicated that SNAP25-deficient smaller boutons contain fewer mitochondria. We also found that the volume of MF boutons, which excludes mitochondrial volumes, was still smaller in tdTom^+^ boutons in *Snap25*-cKO than in those of control brains (tdTom^−^: 12.0 ± 4.8 μm^3^ and tdTom^+^: 16.6 ± 7.3 μm^3^ in control brains; tdTom^−^: 14.5 ± 5.9 μm^3^ and tdTom^+^: 10.1 ± 4.1 μm^3^ in *Snap25*-cKO brains, mean ± SD, *n* = 20 boutons from two brains for each genotype, *p* < 0.05, Figure 6H), indicating that not only the reduced number of mitochondria but also other intracellular components contributed to the reduced volume of SNAP25-deficient boutons. Overall, *Snap25* knockout led to the accumulation of abnormally sized vesicles but did not affect the mitochondrial volume or density in MF boutons.

## Discussion

The present study demonstrated that the conditional knockout of *Snap25* in granule cells of the hippocampal dentate gyrus does not prevent synapse formation but induces a decrease in the size of MF boutons and impairs the formation of dendritic excrescences on CA3 pyramidal neurons. These results indicate that SNAP25 in presynaptic MF boutons is necessary for the morphogenesis of both presynaptic and postsynaptic specialized structures. Our earlier study demonstrated that presynaptic SNAP25 in layer 5 corticothalamic projections is essential for bouton formation and dendritic morphogenesis in the Po of the thalamus.^13^ Taken together, these findings suggest that SNAP25-dependent synaptic morphogenesis is a common mechanism underlying the formation of complex synaptic specialization in layer 5 corticothalamic projections and hippocampal MF-CA3 synapses.

A previous study reported that *Snap25*-cKO brains do not exhibit noticeable defects in MF boutons.^30^ The discrepancy in the findings between our study and the previous investigation might have stemmed from the different Cre deliveries that were used. Gustus et al. used retroviral injection to deliver Cre-recombinase to adult hippocampal progenitors, whereas our study used the genetic expression of *Rbp4-Cre*, which becomes detectable in the first postnatal week in a subset (approximately 40%) of granule cells.^29^^,^^34^^,^^35^ We observed that in the *Rbp4-Cre*^+^ (tdTom^+^) bouton population, *Snap25* deletion was associated with a significant reduction in bouton size at both the light microscopy and EM levels. Given the reportedly high variability in MF bouton size,^43^^,^^44^ it may be difficult to detect a subtle reduction in bouton size when a relatively small, randomly selected sample from the entire MF population is analyzed. It is also possible that the effects of *Snap25* deletion are different between developmentally born granule cells and adult-born cells. Importantly, even when morphological differences in MF boutons were difficult to detect, the labeling of postsynaptic neurons via *in utero* electroporation allowed us to identify the failed formation of dendritic excrescences at the light microscopy level. Furthermore, 3D-CLEM revealed ultrastructural defects in the contact surface of SNAP25-deficient MF, thus indicating that these postsynaptic labeling and 3D ultrastructural analyses are powerful tools for analyzing morphological changes in complex synaptic specialization.

In the absence of SNAP25, neurotrophic factor secretion can be suppressed in addition to synaptic transmission. SNAP25 controls the release of brain-derived neurotrophic factor from the axons and dendrites of cortical neurons.^45^ A recent study revealed that the deletion of *Ntf3*, which encodes the neurotrophic factor neurotrophin-3, in dentate gyrus granule cells causes both the reduced formation of thorny excrescences in CA3 dendrites and functional defects in memory.^46^ This finding supports the idea that the presynaptic vesicular release of neurotrophic factors is essential for the morphogenesis of postsynaptic excrescence formation. However, conflicting evidence also exists; the suppression of vesicular release by *Emx1*-driven tetanus toxin, a protease that cleaves the SNARE complex protein vesicle-associated membrane protein 2 (VAMP2, also known as synaptobrevin-2), increases the density and volume of excrescences in MF-CA3 synapses.^33^ Although both VAMP2 and SNAP25 are necessary for brain-derived neurotrophic factor release at the neurites of cortical neurons,^45^ a previous study showed that evoked neurotransmitter release is more defective in cultured hippocampal neurons from *Snap*25 KO mice than in those from *Vamp2* KO mice.^47^ These differential effects of evoked neurotransmitter release might be related to the different phenotypes of SNAP25-deficient and VAMP2-deficient mice in MF-CA3 synapses. In addition, deletion of the kainate receptor subunit GluK2 leads to the delayed maturation of MF-CA3 synapses,^11^ suggesting the importance of postsynaptic glutamate receptors.

The interaction between presynaptic and postsynaptic neurons is mediated by surface proteins and is crucial for axon targeting and synaptic development/maintenance.^4^^,^^48^ In MF-CA3 synapses, the postsynaptic protein ligand of Numb protein X1 regulates the targeting of MFs through interactions with erythropoietin-producing-hepatocellular B receptors and the subsequent maturation of synapses.^49^ Another study demonstrated that the cell polarity protein Vang-like protein 2 (VANGL2) is expressed in both MFs and CA3 regions, and *Vangl2*-cKO driven by *Emx1-Cre* results in the delayed maturation of MF boutons with reduced volume, surface area, and complexity of thorny excrescences.^50^ Additionally, VANGL2 associates with the heparan sulfate proteoglycan glypican 4, which is essential for organizing synaptic architecture through its interaction with the postsynaptic G-protein-coupled receptor-like orphan receptor 158 (GPR158).^51^ In *Gpr158*-KO mice, MF synapses on CA3 proximal dendrites in the stratum lucidum are increased, whereas spines on CA3 distal dendrites, which receive inputs from other CA3 axons in the stratum radiatum, are unaffected, suggesting that glypican 4-GPR158 is a dendritic region-specific regulator of synapse formation/maintenance.^51^ In *Snap25*-cKO brains, excrescences were normally formed in wild-type (*Rbp4-Cre*^−^) boutons adjacent to SNAP25-deficient (*Rbp4-Cre*^+^) boutons that lacked excrescences from the same dendrites. Although the knockout effects on excrescence formation differ between *Gpr158* and *Snap25*, both proteins function in an input-specific manner along the apical dendrites of CA3 pyramidal neurons, suggesting that the morphogenesis of MF-CA3 synapses requires region (or layer)-specific and individual presynapse-specific regulation.

What are the implications of losing the complex synaptic connections between MFs and CA3 neurons? Previous studies using mouse models of neuropsychiatric diseases have identified links between altered MF structure and abnormal synaptic transmission.^18^ In the transchromosomic Tc1 mouse model of Down syndrome, which carries most of human chromosome 21, the perforation of thorny excrescences is decreased and short-term plasticity and spatial working memory are impaired.^52^ An ultrastructural analysis using SBF-SEM revealed that in a mouse model of familial Alzheimer disease, the complexity and synaptic contact of thorny excrescences are decreased.^53^ In these mutant mice, the synaptic transmission and plasticity of MFs are also altered. Although it is not feasible to record the electrical properties of *Snap25*-cKO synapses because of suppressed synaptic transmission in the absence of regulated vesicular release, the aforementioned studies of mouse models of human disease suggest that the failure to form connections between MF boutons and thorny excrescences of CA3 dendrites can lead to reduced synaptic transmission and impaired hippocampal functions. *Snap25* knockout in cortical layers 5, 6a, and 6b exhibits different onsets of degeneration; layer 5 callosal and corticofugal projections are the earliest of the three to manifest degenerative features.^27^ Unlike callosal and subcortical projections from cortical layer 5, there was no apparent degeneration in SNAP25-deficient MF boutons under fluorescence microscopy. However, compared with control boutons, these boutons were more likely to have dark cytoplasm and large clear synaptic vesicles. Previous studies have reported that degenerating axon terminals have an electron-dense cytoplasm.^54^^,^^55^ Such altered intracellular profiles have also been observed in the cerebral cortex of *Snap25*-cKO mice.^27^ Although MF boutons contain large clear vesicles even under physiological conditions,^9^^,^^56^ these vesicles are increased in the cerebral cortical neurons from Alzheimer disease patients.^41^ These findings suggest that intracellular alterations in *Snap25*-cKO MF boutons may be associated with presynaptic degeneration.

In summary, the present study indicates that presynaptic SNAP25 is essential for the morphogenesis of synaptic specialization in MF-CA3 synapses. The morphological defects and intracellular alterations observed in the MF-CA3 synapses of *Snap25*-cKO mice provide insights into the synaptic mechanisms underlying neuropsychiatric and degenerative disorders.

### Limitations of the study

Researchers have used a range of silencing techniques in different brain regions to investigate the role of neuronal activity in synaptic development.^13^^,^^31^^,^^32^^,^^33^ Our findings are consistent with those of earlier studies in suggesting that the initial formation of synapses occurs independently of presynaptic input. However, the effects of silencing varied in the subsequent synaptic maturation, including postsynaptic morphogenesis. Further research is necessary to identify which regulatory processes are influenced by each genetic manipulation. The current study has three main limitations. First, we failed to distinguish whether the phenotype reflected the loss of neurotransmitter release, neurotrophin release, another SNAP25-dependent secretory pathway, or secondary degenerative changes. Second, our work is primarily structural, and functional assessment is required in MF-CA3 transmission, plasticity, or hippocampal behavior of the *Snap25*-cKO model. Third, conditional knockout of *Snap25* using *Rbp4-Cre* is not temporally inducible, and this has hindered the distinction between the developmental and maintenance roles of SNAP25. If a mouse line with inducible Cre that is specifically expressed in the dentate gyrus becomes available, it will be useful for determining how the failed maturation of MF-CA3 synapses is related to their degeneration in the absence of SNAP25.

## Resource availability

### Lead contact

Requests for further information and resources should be directed to and will be fulfilled by the lead contact, Shuichi Hayashi (s.hayashi@med.kawasaki-m.ac.jp).

### Materials availability

This study did not generate new unique reagents.

### Data and code availability

• The data reported in this study are available from the lead contact upon request.
• No original code was developed for the analysis.
• Any additional information required to reanalyze the data reported in this paper is available from the lead contact upon request.

## Acknowledgments

We thank N. Hattori and A. Imai for their technical assistance with SBF-SEM sample preparation and data handling. We also thank Bronwen Gardner, PhD, from Edanz (https://jp.edanz.com/ac) for editing a draft of this manuscript. The SBF-SEM sample preparation and data acquisition were supported by the Cooperative Study Programs of the National Institute of Physiological Sciences (NIPS). SH was supported by the 10.13039/501100001691Japan Society for the Promotion of Science (JSPS) Grants-in-Aid for Scientific Research (10.13039/501100001691KAKENHI) (19K23786 and 22K06242), 10.13039/100007449Takeda Science Foundation, 10.13039/501100013171Ryobi Teien Memory Foundation, 10.13039/100016189Wesco Scientific Promotion Foundation, and 10.13039/501100009158Kawasaki Medical School Research Project. Z.M. was supported by a 10.13039/501100000265Medical Research Council (MRC) Project Grant (G00900901) and a St John’s Research Centre Grant (21138077).

## Author contributions

Conceptualization, methodology, and writing – original draft, S.H., N.O., and Z.M.; investigation, formal analysis, and visualization, S.H. and N.O.; supervision, K.T.

## Declaration of interests

The authors declare no competing interests.

## STAR★Methods

### Key resources table

**Table:** 

REAGENT or RESOURCE	SOURCE	IDENTIFIER
**Antibodies**		

guinea pig anti-VGLUT1	Merck	Cat# AB5905; RRID: AB_2301751
chicken anti-GFP	Invitrogen	Cat# A10262; RRID: AB_2534023
rabbit anti-HOMER1	Synaptic Systems	Cat# 160 003; RRID: AB_887730
guinea pig anti-Bassoon	Synaptic Systems	Cat# 141 005; RRID: AB_2924946
DyLight405-conjugated donkey anti-rabbit IgG (H + L)	Jackson ImmunoResearch	Cat# 711-475-152; RRID: AB_2340616
Fluorescein (FITC)-conjugated donkey anti-chicken IgY (H + L)	Jackson ImmunoResearch	Cat# 703-095-155; RRID: AB_2340356
Cy5-conjugated donkey anti-guinea pig IgG (H + L)	Jackson ImmunoResearch	Cat# 706-175-148; RRID: AB_2340462

**Plasmid**		
pCAG-EYFP	Hayashi et al., 2017	N/A

**Experimental models: Organisms/strains**		

Mouse: *Rbp4-Cre*	The Mutant Mouse Resource & Research Center	Tg(Rbp4-cre)KL100Gsat/Mmucd
Mouse: Ai14	The Jackson Laboratory	B6;129S6-*Gt(ROSA)26Sortm14(CAG-tdTomato)Hze/J*
Mouse: *Snap25-flox*	Wilson lab	B6-*Snap25tm3mcw*

**Oligonucleotides**		

*LoxP* E5a F	IDT	ccctggggaaccacggcaga
*LoxP* E5a R	IDT	tcccaggaaacagcacagcgt

**Software and algorithms**		

Fiji	Schindelin et al., 2012	https://imagej.net/software/fiji/
VAST Lite	Lichtman lab	https://lichtman.rc.fas.harvard.edu/vast/
Blender 4.0	Blender Foundation	https://www.blender.org/download/releases/4-0/
Prism 8	GraphPad	https://www.graphpad.com/features

GFP, green fluorescent protein; HOMER1, homer scaffolding protein 1; IgG, immunoglobulin G; VGLUT1, vesicular glutamate transporter 1.

### Experimental model and study participant details

#### Animals

Animal experiments were conducted in the animal facility at Kawasaki Medical School with the approval of the Animal Research Committee of Kawasaki Medical School (21–047, 23–071, and 24–041). All experiments were performed in accordance with the “Guide for the Care and Use of Laboratory Animals” of the Kawasaki Medical School. Tg(Rbp4-cre)KL100Gsat/Mmucd (*Rbp4*-Cre, The Mutant Mouse Resource & Research Center, MMRRC) mice were crossed with B6;129S6-*Gt(ROSA)26Sortm14(CAG-tdTomato)Hze/J* (Ai14, The Jackson Laboratory) mice to label neurons in the dentate gyrus of the hippocampus. To generate *Rbp4-Cre;Ai14;Snap25*^*fl/fl*^ mice, these mice were then crossed with B6-*Snap25tm3mcw* (*Snap25*^*fl/fl*^) mice, which were obtained from the University of New Mexico (Michael C. Wilson).^27^ Both male and female mice were used in the experiments using optical microscopes (Figures 1, 2, S1, and S2), and male mice were used in the experiments using SBF-SEM (Figures 3, 4, 5, and 6).

### Method details

#### In utero electroporation

*In utero* DNA transfer to the hippocampal CA3 by electroporation was performed as previously described.^57^ Briefly, for EYFP overexpression, 1–2 μL of phosphate-buffered saline (PBS) containing 1.0 mg/mL of pCAG-EYFP was introduced into the left ventricle of the brain at E15.5. Next, five 50-ms pulses of 35 V were delivered to the embryonic heads at 950-ms intervals using electrodes (CUY650P5, NEPAGENE) connected to an electroporator (NEPA21 Type II, NEPAGENE). The brains were fixed at P21, 6 weeks of age, and adult (3–4 months old) time points.

#### Immunostaining and imaging

Young animals (aged 3–6 weeks) and adults (aged 3–4 months) were deeply anesthetized and perfusion-fixed with 4% paraformaldehyde (Sigma-Aldrich, 81715) in 0.1 M phosphate buffer. The brains were post-fixed in the same solution for 16–20 h at 4 °C. Using a vibrating microtome (Leica Microsystems, VT1200S), the brains were coronally sectioned at 50 μm. Sections containing the dorsal hippocampus were then incubated in a blocking solution containing PBS with 1% bovine serum albumin and 0.3% Triton (blocking solution) for 2 h, followed by overnight incubation at 20 °C with primary antibodies diluted in blocking solution. Staining was visualized by incubation with secondary antibodies for 2 h at 20 °C. The sections were counterstained with Hoechst 33342 (0.4 μg/mL, Sigma-Aldrich, B2261) to visualize the nuclei. Primary antibodies were as follows: guinea pig anti-VGLUT1 (Merck, AB5905, 1:2,000), chicken anti-GFP (Invitrogen, A10262, 1:5,000), and rabbit anti-HOMER1 (Synaptic Systems, 160 003, 1:1,000). The following secondary antibodies (Jackson ImmunoResearch) were used at 1:1,000 dilution: fluorescein-conjugated donkey anti-chicken immunoglobulin (Ig)Y (H + L) (703-095-155), Cy5-conjugated donkey anti-guinea pig IgG (H + L) (706-175-148), and DyLight405-conjugated donkey anti-rabbit IgG (H + L) (711-475-152).

#### CLEM

The CLEM workflow was performed as previously described,^37^^,^^38^ with some modifications to analyze hippocampal MF boutons in the CA3 region.^39^ Briefly, brains from 3- to 4-month-old *Rbp4-Cre;Ai14;Snap25*^*+/f*^ and *Rbp4-Cre;Ai14;Snap25*^*fl/fl*^ mice were perfused with 0.1 M phosphate buffer containing 2% paraformaldehyde (Electron Microscopy Sciences, 15714) and 2.5% glutaraldehyde (Electron Microscopy Sciences, 16220) at pH 7.4, and were then post-fixed at room temperature for 2 h. The dissected brains were sectioned on a vibratome at 80 μm, and fluorescence and bright-field images of hippocampal regions at various magnifications were collected using an epifluorescence microscope (BX61TRF, Olympus) and a confocal microscope (LSM700, Zeiss). The positions of tdTom^+^ boutons in the lateral half of CA3 (approximately in CA3a and the lateral half of CA3b) were imaged together with major blood vessels as fiducial marks so that boutons in fluorescence images were able to be localized in electron micrographs by their locations with respect to these features. The sections were then post-fixed in 1.5% potassium ferrocyanide (FUJIFILM Wako, 163–03742) and 2% osmium tetroxide (Nisshin EM, 3020) in PBS. They were then stained with 1% thiocarbohydrazide (Sigma-Aldrich, 223220) followed by 2% osmium tetroxide and overnight staining with 2% uranyl acetate. The final stain was at 50 °C for 2 h in a lead aspartate solution (pH 5–5.5). Next, the sections were washed in water, dehydrated in a graded ethanol series (60%, 80%, 90%, and 95% for 5 min each), infiltrated with acetone, and then infiltrated with 1:1 and 1:3 mixtures of acetone and Durcupan resin, which was mixed according to the manufacturer’s instructions (Sigma-Aldrich, 44611, 44612, 44613, 44614), before being infiltrated with 100% resin. The sections were mounted at the bottom of a flat embedding mold, and the resin was hardened at 60 °C for 2 nights. The samples were then imaged using a scanning electron microscope (Merlin, Zeiss) equipped with a 3View cutting system (Gatan). The size of the obtained stacks was as follows: *xy*, 75–80 μm each (6 nm/pixel); *z*, 20–30 μm (30-nm interval). The obtained image series were aligned using the alignment functions of the TrakEM2 plugin for Fiji (https://imagej.net/software/fiji/).^58^ SBF-SEM images were correlated with fluorescence images using tissue landmarks, such as blood vessels and cell bodies, and tdTom^+^ boutons were identified in the SBF-SEM images. The tdTom^+^ boutons and their connecting dendrites were segmented using VAST Lite (https://lichtman.rc.fas.harvard.edu/vast/). The segmentation of boutons and dendrites was exported in OBJ file format and imported into Blender 4.0 (https://www.blender.org/download/releases/4-0/) for 3D reconstruction. Mesh models were created from the selected boutons and dendrites, including excrescences, and their volumes and surface areas were measured in Blender using the volume and surface area measurement functions of the NeuroMorph tools.^59^ MF boutons were defined as regions in which the axonal shaft enlarges, contacts dendritic shafts or thorny excrescences, and contains a high density of synaptic vesicles. Synaptic sites were defined as contact sites between MF boutons and dendritic shafts or thorny excrescences with the following structures: increased synaptic vesicle density, touching of some vesicles to the presynaptic membrane, a cleft between the presynaptic and postsynaptic membranes, and an asymmetric thickening of the postsynaptic membrane. However, the latter two structures were not always visible in the samples used in the present study.

### Quantification and statistical analysis

Fiji software was used to analyze the fluorescence microscopy and EM images. Quantification was performed by researchers blinded to the mouse strain. In the quantitative analysis using fluorescent microscopy, one slice containing EYFP-positive neurons in the CA3 region was selected for each brain, and a total of 30 boutons (10 boutons per brain, total three brains) were analyzed for each of control and *Snap25*-cKO brains. To quantify bouton density, tdTom^+^ and VGLUT1^+^ boutons within a volume of 2,000 μm^3^ (*xy*: 20 μm × 20 μm, *z*: 5 μm) were counted, and the density (per 1 × 10^3^ μm^3^) was compared between control and *Snap25*-cKO brains. The size of tdTom^+^ boutons and their connecting excrescences was quantified by segmenting tdTom^+^ boutons and EYFP^+^ excrescences on each z stack plane (0.5 μm intervals) using VAST Lite software, and the total segmented areas (cross-sectional areas) of boutons and excrescences were compared between the control and *Snap25*-cKO brains. For HOMER1 cluster quantification, a consistent threshold value was applied to HOMER1 fluorescence images in Fiji for both control and *Snap25*-cKO boutons, and the number of clusters was measured in each optical plane. Fluorescent signals from the same cluster in adjacent planes were manually excluded when counting HOMER1 clusters. To analyze the intracellular vesicles in the boutons, the central plane of the SBF-SEM image stacks containing the bouton of interest was selected, and vesicle diameter was measured. Mitochondria were manually segmented using VAST-Lite, and 3D models were exported to Blender to analyze MF boutons and excrescences and compare the mitochondrial volume in the boutons. The obtained data were analyzed using the Mann–Whitney *U*-test (Figure 2) or the Kruskal–Wallis test followed by Dunn’s multiple comparison test as a *post hoc* test (Figures 1, 5, and 6) using Prism 8 software (GraphPad).

## Supplemental information

Document S1. Figures S1 and S2

Video S1. SBF-SEM images of MF boutons and their connecting dendrite in a control brain, related to Figure 5 Animation of an ultrastructural image series taken with SBF-SEM from a hippocampal slice of the *Rbp4-Cre;Snap25(f/+);Ai14* (control) brains shown in Figures 5A and 5C. The tdTom^−^ (blue and green) and tdTom^+^ (red) boutons that contacted the same dendrite (yellow) are indicated in different colors. Scale bars: 1 μm.

Video S2. SBF-SEM images of MF boutons and their connecting dendrite in a *Snap25*-cKO brain, related to Figure 5 Animation of an ultrastructural image series taken with SBF-SEM from a hippocampal slice of the *Rbp4-Cre;Snap25(f/f);Ai14* (*Snap25*-cKO) brains shown in Figures 5B and 5E. The tdTom^−^ (light blue and green) and tdTom^+^ (purple, magenta, and pink) boutons that contacted the same dendrite (yellow) are shown in different colors. Scale bars: 1 μm.
