# Supplementary material for: Presynaptic SNAP25 supports maturation of hippocampal mossy fiber-CA3 synapses
Source: iScience. 2026 Jun 20;29(7):116503. doi: 10.1016/j.isci.2026.116503 (PMC13315434; doi:10.1016/j.isci.2026.116503)
Supplement: Document S1. Figures S1 and S2 [file mmc1.pdf]

iScience, Volume *29*

Supplemental information

Presynaptic SNAP25 supports maturation of hippocampal mossy fiber-CA3 synapses

Shuichi Hayashi, Nobuhiko Ohno, Zoltán Molnár, and Kazunori Toida

Supplementary information

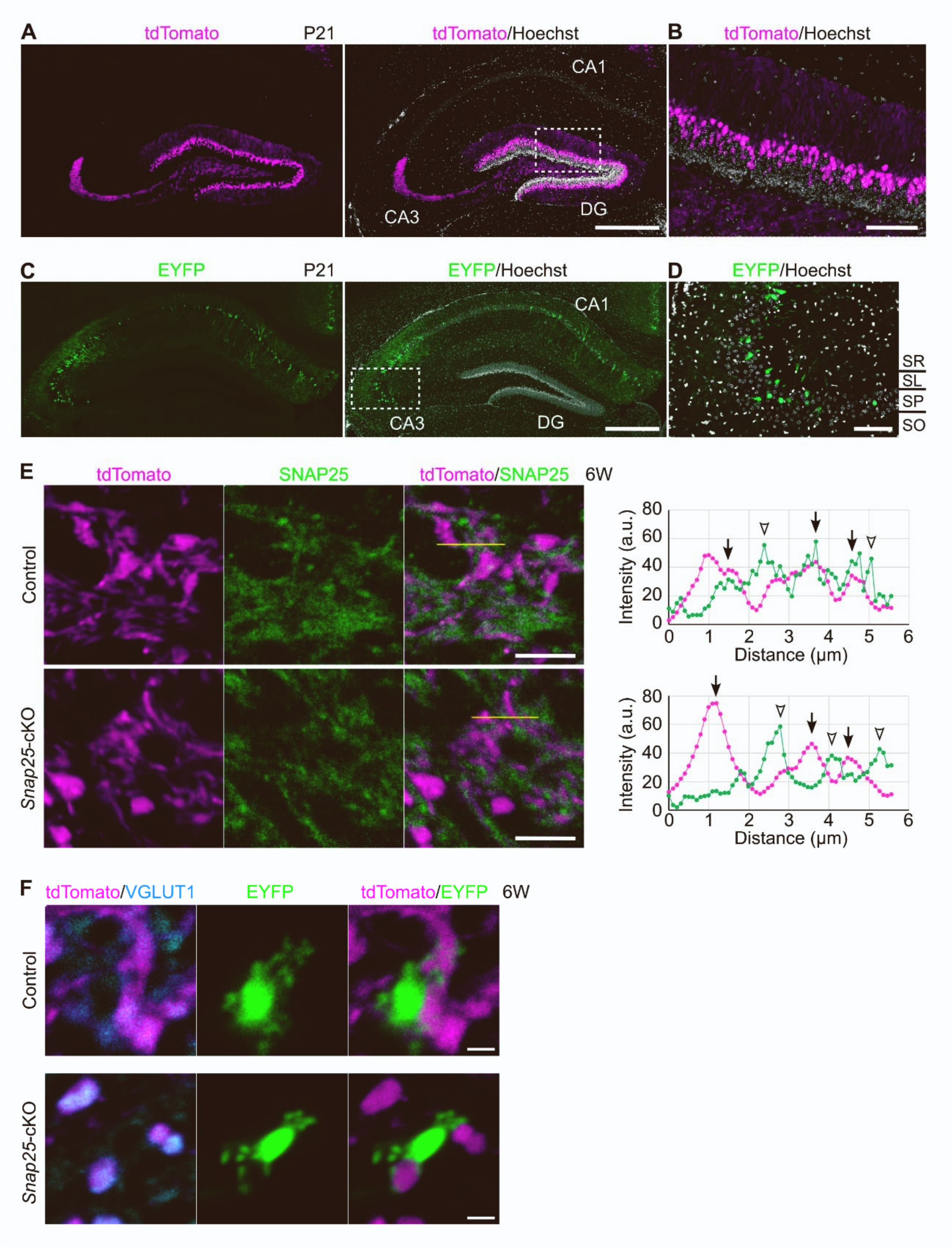

Figure S1. Labeling of MFs and CA3 dendrites with different fluorescent proteins, related to Figure 1.

(A, B) Expression of tdTomato fluorescence in the hippocampus. tdTomato was expressed in a subset of granule cells in the dentate gyrus, and their axons extended into the CA3 region. (B) shows an enlarged image of the boxed region in (A). DG, dentate gyrus; CA, cornu ammonis.

(C, D) Neurons in CA1, 2, and 3 were sparsely labelled with EYFP by *in utero* electroporation. (D) shows an enlarged image of the boxed region in (C). SR, stratum radiatum; SL, stratum lucidum; SP, stratum pyramidale; SO, stratum oriens.

(E) SNAP25 staining of MFs in the CA3 region. The right graphs show a line scan of the fluorescence intensity (arbitrary units, a.u.) along the yellow lines in the left images. Black arrows indicate tdTomato fluorescence peaks. White arrowheads indicate the peaks of SNAP25 signals in the $tdTom^{-}$ fibers. tdTomato was colocalized with SNAP25 in control brains but not in *Snap25*-cKO brains.

(F) Examples of MF boutons that contacted CA3 dendrites labeled with EYFP at 6 weeks of age. Thorny excrescences from the connecting CA3 dendrite extended into the control MF bouton (upper panel). Dendritic branches only contacted the outside of the connecting *Snap25*-cKO boutons (lower panel).

Scale bars: 500 μm in (A, C); 100 μm in (B, D); 5 μm in (E); 2.5 μm in (F).

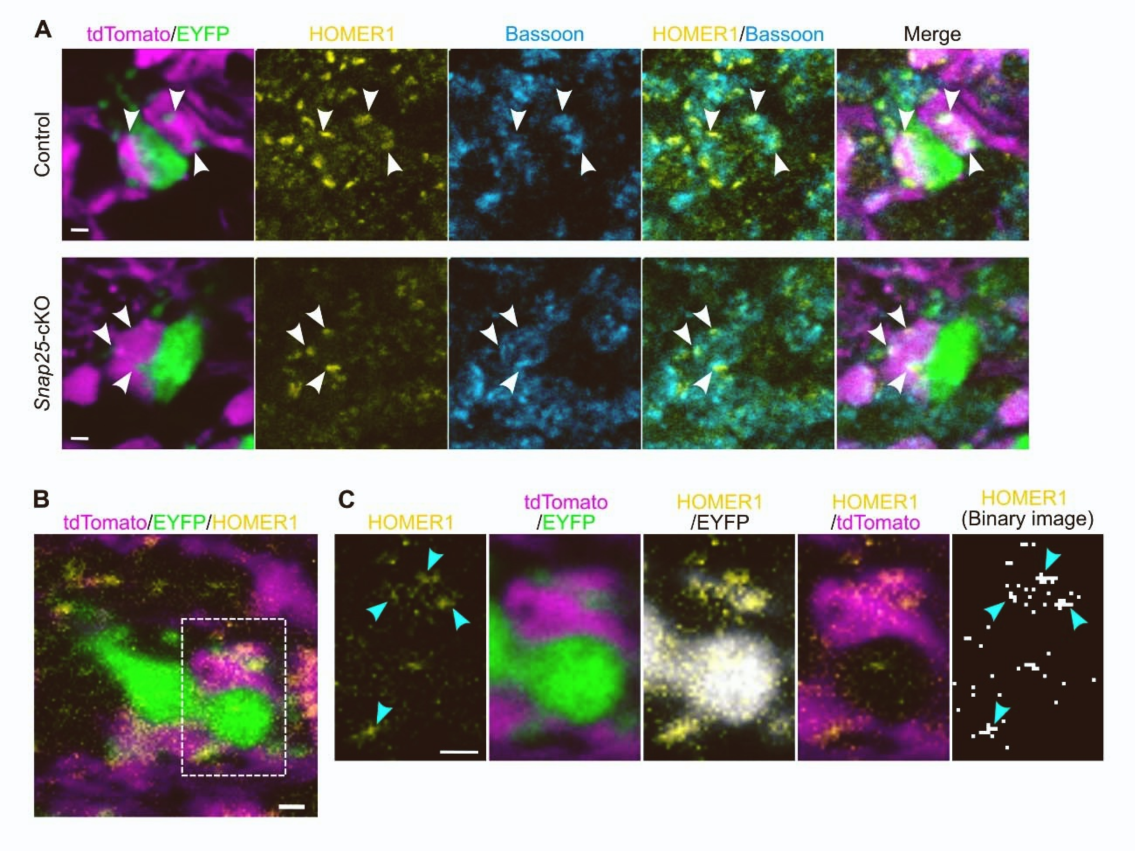

Figure S2. Localization of HOMER1 clusters on EYFP-labelled excrescences of CA3 dendrites that connect with tdTom$^+$ boutons, related to Figure 2.

(A) In both control and SNAP25-deficient boutons at 6 weeks of age, HOMER1 and Bassoon were assembled at the contact sites between tdTom$^+$ boutons and EYFP-labelled CA3 dendrites (arrowheads).

(B) A typical example image showing HOMER1 localization on an EYFP-positive CA3 dendrite that contacts tdTom$^+$ boutons in a control brain at 6 weeks of age. (C) Enlarged image of the boxed region in (B). Blue arrowheads indicate examples of HOMER1 clusters on excrescences that contact tdTom$^+$ boutons. The right image shows a binary image of HOMER1 for measuring the area of the clusters. The signals from a single pixel were excluded from the measurements. Also see the STAR Method section for more details of the analysis. Scale bars, 1 µm.
